# A New F131V Mutation in Chlamydomonas Phytoene Desaturase Locates a Cluster of Norflurazon Resistance Mutations near the FAD-Binding Site in 3D Protein Models

**DOI:** 10.1371/journal.pone.0099894

**Published:** 2014-06-17

**Authors:** Julio V. Suarez, Stephen Banks, Paul G. Thomas, Anil Day

**Affiliations:** 1 Faculty of Life Sciences, The University of Manchester, Manchester, United Kingdom; 2 Syngenta, Bracknell, Berkshire, United Kingdom; University of South Florida College of Medicine, United States of America

## Abstract

The green alga *Chlamydomonas reinhardtii* provides a tractable genetic model to study herbicide mode of action using forward genetics. The herbicide norflurazon inhibits phytoene desaturase, which is required for carotenoid synthesis. Locating amino acid substitutions in mutant phytoene desaturases conferring norflurazon resistance provides a genetic approach to map the herbicide binding site. We isolated a UV-induced mutant able to grow in very high concentrations of norflurazon (150 µM). The phytoene desaturase gene in the mutant strain contained the first resistance mutation to be localised to the dinucleotide-binding Rossmann-likedomain. A highly conserved phenylalanine amino acid at position 131 of the 564 amino acid precursor protein was changed to a valine in the mutant protein. F131, and two other amino acids whose substitution confers norflurazon resistance in homologous phytoene desaturase proteins, map to distant regions in the primary sequence of the *C. reinhardtii* protein (V472, L505) but in tertiary models these residues cluster together to a region close to the predicted FAD binding site. The mutant gene allowed direct 5 µM norflurazon based selection of transformants, which were tolerant to other bleaching herbicides including fluridone, flurtamone, and diflufenican but were more sensitive to beflubutamid than wild type cells. Norflurazon resistance and beflubutamid sensitivity allow either positive or negative selection against transformants expressing the mutant phytoene desaturase gene.

## Introduction

*Chlamydomonas reinhardtii* provides an attractive model organism to dissect processes unique to photosynthetic eukaryotes [1]. Forward genetic screens for gain-of-function mutations that confer herbicide resistance provide a powerful approach to investigate herbicide mode of action [2]. These studies proceed rapidly using algal and plant models containing herbicide target sites, which are often absent in animals and fungi. Resistance resulting from a mutation in a target protein can identify an herbicide’s binding site, which is normally revealed by identifying and sequencing the corresponding mutant gene. Identifying genes responsible for herbicide resistance is facilitated by the availability of complete sequences of the *C. reinhardtii* nuclear and organelle genomes [3], [4]. Forward genetics provided a powerful tool to locate the binding sites of triazine herbicides in the D1 protein of photosystem II by sequencing mutant *psbA* genes encoding D1 from herbicide tolerant *C. reinhardtii* strains [5], [6], [7]. The locations of the amino acid substitutions in the D1 protein identified the herbicide target site which overlapped with the Q_B_ quinone binding site in photosystem II [8], [9].

Norflurazon (CID 33775) is a bleaching herbicide that blocks the synthesis of carotenoids by inhibiting the activity of phytoene desaturase (PDS) [10]. The first committed step of the carotenoid synthesis pathway produces phytoene in a reaction catalysed by phytoene synthase. PDS catalyses the next step of the pathway involving the two-step dehydrogenation of phytoene to produce zeta-carotene via a phytofluene intermediate [11], [12]. Carotenoids protect chloroplasts from excess light energy [13], [14] and their absence results in chlorophyll bleaching and eventual cell death [15]. In *C. reinhardtii*, PDS is encoded by the single nuclear *PDS1* gene (Cre12.g509650) located on chromosome 12 [4]. The *C. reinhardtii* and *A. thaliana* PDS3 (At4G14210.1) proteins are homologous and share 66% amino acid identity.

PDS mutations conferring norflurazon resistance have been described in algae, plants and cyanobacteria. A *C. reinhardtii* mutant tolerant to 2 µM norflurazon has been described [16] but the mutation (s) responsible remain uncharacterised. Substitutions at arginine 304 (R304S, R304T, R304H, R304C) of the 580 amino acid PDS protein from the aquatic flowering plant *Hydrilla verticillata* conferred resistance to norflurazon and the related bleaching herbicide fluridone [17], [18]. A L516F substitution in the 558 amino acid PDS protein of the green alga *Chlorella zofingiensis* prevented bleaching in media containing 0.25 µM norflurazon; wild type (WT) cells were bleached by 0.05 µM norflurazon [19]. In the cyanobacterium *Synechococcus* PCC 7942, mutant PDS proteins conferring resistance to norflurazon contain R195P, L320P, V403G and L436R substitutions [11]. The herbicide lethal dose was 40 µM and 70 µM for strains harbouring the R195P and L436R substitutions, respectively, and 0.5 µM for WT *Synechococcus* [11]. The 474 amino acid *Synechococcus* and *C. reinhardtii* PDS proteins share 62% identity over 448 amino acids. In Synechocystis PCC 6803, R195P and R195S substitutions in PDS gave higher levels of norflurazon resistance than a R195C substitution [20]. The relationship between these resistance mutations located at distant sites of the plant, algal and cyanobacterial PDS proteins and the norflurazon target site in the folded protein is not known.

*C. reinhardtii* mutants tolerant to norflurazon have been previously isolated [16], [21] but the mutant genes responsible for herbicide tolerance have not been isolated and sequenced. Down regulation of PDS RNA levels by about 80% did not appear to affect carotenoid levels in *C. reinhardtii* [22]. Whilst there are no previous reports of using forward genetics to isolate and sequence mutant PDS alleles conferring norflurazon resistance in *C. reinhardtii*, substitutions that impair PDS function resulting in light-induced bleaching have been identified [23]. Selection with relatively high concentrations of herbicide has the potential to isolate a new *pds1* allele encoding an extremely resistant mutant protein that could act as a marker gene for nuclear transformation [24]. New missense mutations located in regions of the primary sequence not previously associated with herbicide resistance are particularly valuable for mapping the norflurazon binding site. Here we describe the isolation of a new mutant *pds1* allele containing the first herbicide-resistance mutation to be localised to the dinucleotide-binding Rossmann-like domain. The mutant allele confers resistance to 150 µM norflurazon allowing direct herbicide-based selection of nuclear transformants. We mapped this new mutation, and other amino acid substitutions conferring norflurazon resistance in homologous proteins, from algae, cyanobacteria and plants, on 3D models of *C. reinhardtii* PDS to identify the target site of norflurazon.

## Results

### Isolation of a Mutant Strain Highly Resistant to Norflurazon

**Figure 1:**
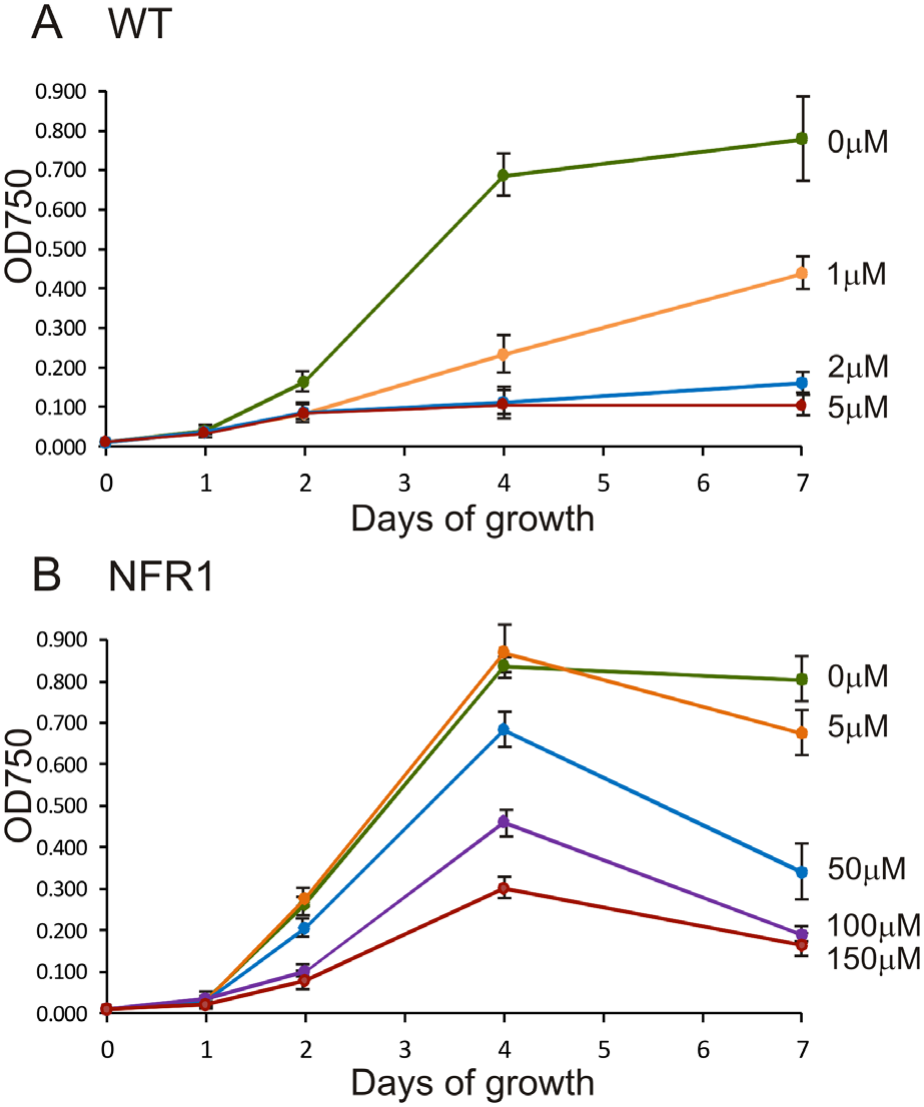
The influence of increasing norflurazon concentration on growth of *C. reinhardtii*. Cultures were inoculated to a density of 1×10^5^ cells/ml on day 0 and optical density at 750 nm used to estimate cell density. (**A**) WT progenitor *PDS1* strain. (**B**) NFR1 mutant *pds1-nfr1d* strain. The mean values of three experiments were plotted. Standard error bars are shown.

**Figure 2:**
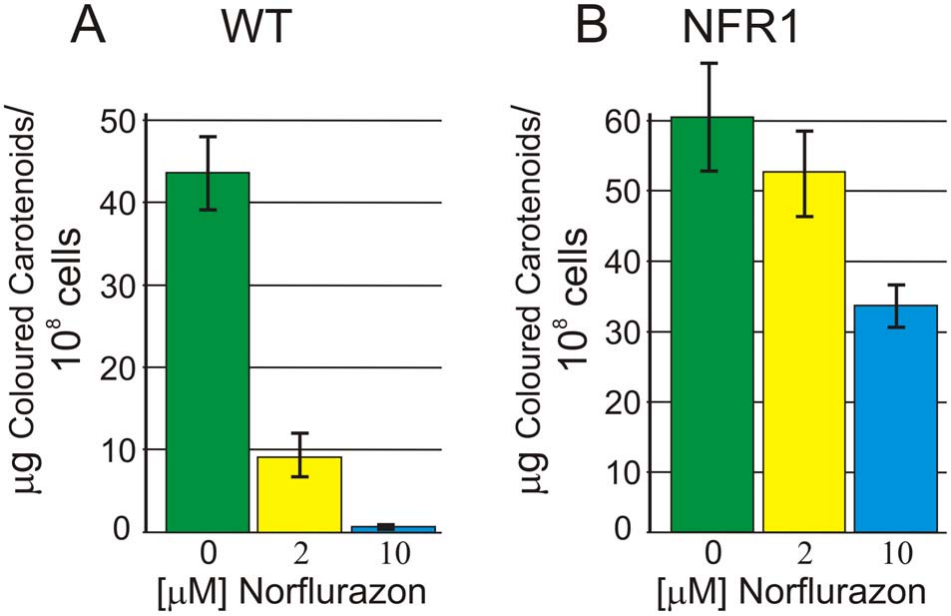
Impact of norflurazon concentration on coloured carotenoid content. (**A**) WT progenitor *PDS1* strain, (**B**) NFR1 mutant *pds1-nfr1d* strain. Data obtained after seven days of growth in a 12 h light/12 h dark cycle from a starting cell density of 10^5^ cells/ml. Norflurazon was added at day 0. Average values of three replicate cultures were plotted. Standard error bars are shown.

After exposure of *C. reinhardtii nic7 arg7 y1* mt+ cells to high energy UV light (254 nm), colonies were selected by their ability to grow on TAP medium containing 33 µM norflurazon. Norflurazon resistant mutant strain 1 (NFR1) was chosen for further analysis because it grew much more strongly under 33 µM herbicide selection. The parent *nic7 arg7 y1* mt+ strain giving rise to NFR1 was sensitive to norflurazon and henceforth will be referred to as wild type (WT). The lowest concentration of norflurazon tested (1 µM) reduced WT cell densities by about 70% after four days of growth in liquid TAP medium (Fig. 1A). Growth of the WT strain was prevented by 5 µM norflurazon. The effective concentration of norflurazon inhibiting WT growth by 50% (Ec50) after a four day period was 0.7±0.1 µM. In contrast, strain NFR1 grew to similar densities in the presence or absence of 5 µM norflurazon after four days growth (Fig. 1B). The NFR1 culture densities were highest at four days when exposed to herbicide and then gradually declined. The Ec50 for the NFR1 strain was calculated to be 108 µM norflurazon at four days, which is over 150-fold higher than the WT Ec50. NFR1 grew in media containing 150 µM norflurazon, the highest concentration tested, and after four days reached cell densities that were about 30% of those reached in the absence of herbicide (Fig. 1B). Coloured carotenoid content was measured by absorbance at 470 nm. The coloured carotenoid content of WT cells was reduced by 79% and 99% following exposure to 2 µM and 10 µM norflurazon, respectively (Fig. 2A). By comparison 2 µM and 10 µM norflurazon reduced coloured carotenoids in NFR1 cells by 13% and 44%, respectively (Fig. 2B).

### Genetic Analysis of Norflurazon Resistance

Segregation analysis of resistance to norflurazon was performed by crossing NFR1 mt+ with WT norflurazon-sensitive strains S1D2 mt- or CC621 mt- and scoring haploid progeny dissected from the meiotic products of diploid zygotes. Of the 179 colonies derived from random haploid spores dissected from tetrads, 81 were norflurazon resistant. This data fits a 1∶1 segregation pattern for a single Mendelian locus better than the 3∶1 segregation ratio predicted for a resistance trait encoded by two unlinked genes, where only 25% of progeny would be resistant to norflurazon. The mutation conferring herbicide resistance was localised to a *pds1-nfr1d* allele encoding a mutant PDS (see below).

**Figure 3:**
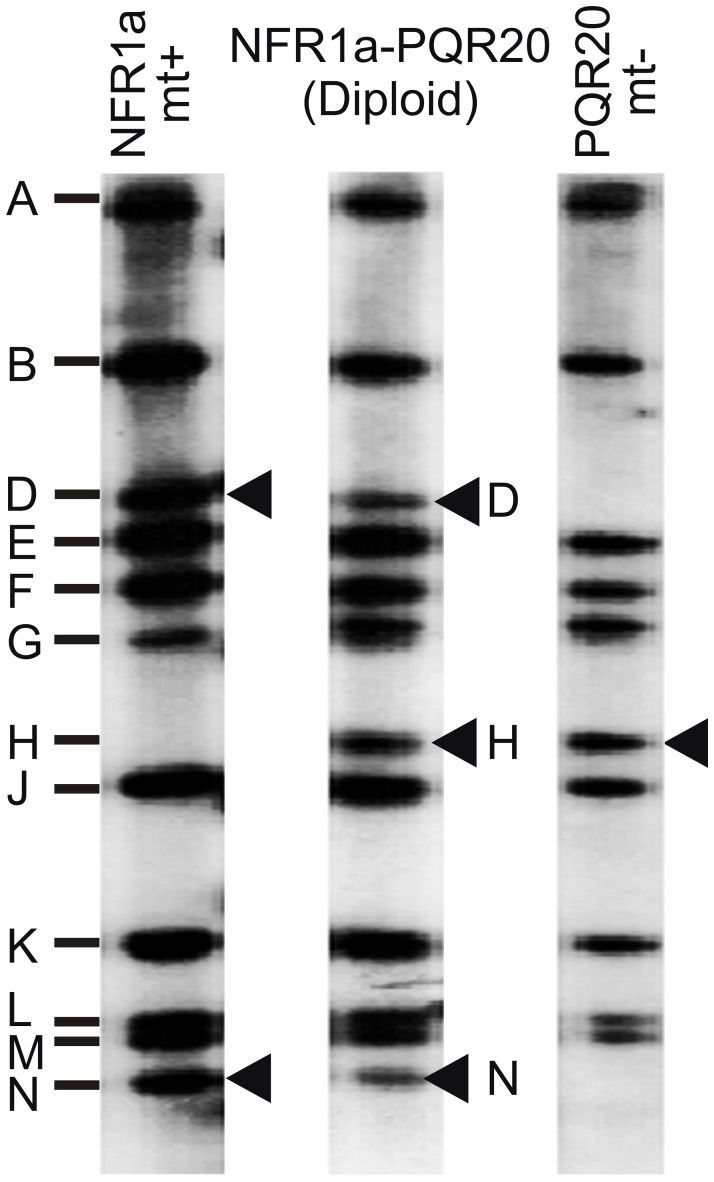
DNA blot showing Gulliver transposon bands in Hind III digests of total DNA from parental NFR1a and PQR20 strains and a diploid strain. Letters on the left represent Gulliver transposons [26]. Arrowheads indicate bands unique to either parent strain.

Diploid (2 n) cells [25] were used to determine whether the mutant allele in the NFR1 strain was dominant or recessive to the corresponding WT gene. Norflurazon resistant strain NFR1a (*pds1-nfr1d*, *arg7* mt+) was mated to a paraquat (PQR) resistant strain PQR20 (*PDS1*, *nic7, pqr* mt−) previously isolated in our laboratory (unpublished) to produce diploid cells. Complementation of the *arg7* and *nic7 loci* in diploid cells resulted in prototrophic growth. All diploid colonies isolated were tolerant to norflurazon and paraquat. This indicated that the mutant genes responsible for norflurazon and paraquat resistance were dominant or semi-dominant to their corresponding WT alleles. Dominance or semi-dominance of the *pds1-nfr1d* allele over the WT *PDS1* gene was confirmed by its use in transformation experiments (see below). DNA blot analysis was used to show that the cells tolerant to both herbicides were indeed diploid rather than recombinant cells. Hind III digests of DNA from diploid strains gave rise to a pattern of Gulliver transposable elements [26] representative of all the elements present in the parental PQR20 and NFR1a strains. An example is shown in Figure 3. NFR1a mt+ possesses two Gulliver bands (D and N in Figure 3) not present in the PQR20 mt− parent, whereas PQR20 contains Gulliver H, which is not present in DNA digests from strain NFR1a. These monosomic D, H and N single copy Gulliver bands unique to one of the parents were less intense in the diploid cells relative to the duplicated Gulliver bands that were common to both parents. This additive pattern of Gulliver elements in diploid strains was consistent with their derivation from the combined genomes of the two parental strains.

### A Base Substitution in the *pds1-nfr1d* Allele Results in a F131V Missense Mutation

**Figure 4:**
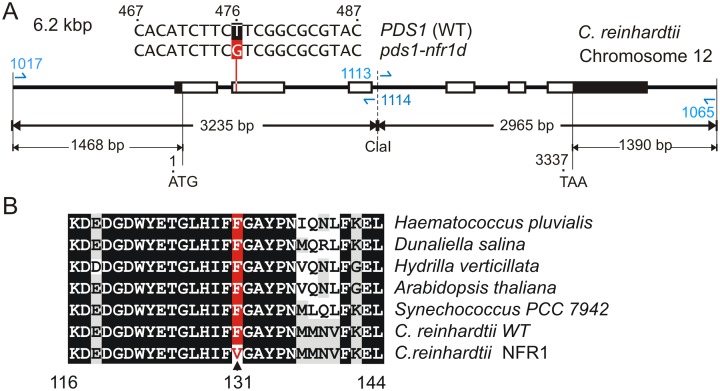
Localisation of the norflurazon resistance mutation in the NFR1 strain. (**A**) Section of Chromosome 12 of *C. reinhardtii* containing the *PDS1* gene (Cre12.g509650). Exons are shown as boxes; white boxes represent coding regions and black boxes UTRs. Base one is the adenine in the ATG start codon. At base 476 in exon 2, the WT thymine is replaced by guanine in the *pds1-nfr1d* allele resulting in a phenylalanine to valine missense mutation. Primers used to amplify the gene and an inserted *Cla I* site are shown. (**B**) The F131V substitution (red) is found within a highly conserved region of the PDS protein. Coordinates correspond to the *C. reinhardtii* PDS sequence, where amino acid one is the initiator methionine at the start of the transit peptide.

The PDS enzyme is a well characterised target of norflurazon [10], [11], [18], [27]. The *PDS1* gene (Cre12.g509650) located on chromosome 12 is comprised of six exons (Fig. 4A) which are spliced together into a continuous coding region of 1692 nucleotides terminated by a TAA stop codon. All the *pds1-nfr1d* exons from strain NFR1 genomic DNA were amplified into PCR products and sequenced directly. Two mutations were found in the *pds1-nfr1d* allele compared to the *C. reinhardtii* WT *PDS1* sequence in GenBank (Acc. No. XM_001690807). A guanine to adenine transition in exon three (base 987 of the CDS) was a silent mutation that does not change glycine 329 in the protein; base one is the adenine in the start ATG codon. A thymine to guanine transversion in exon 2, located at base 476 of the gene (base 391 of the coding region), changed the phenylalanine TTC codon at amino acid position 131 to a valine GTC codon (Fig. 4A). This F131V missense mutation resides within the Rossmann-like domain (see below) in a region showing a high degree of conservation between cyanobacterial, algal and plant PDS proteins (Fig. 4B).

### Use of the Mutant *pds1-nfr1d* Allele as a Selectable Marker for Transformation

**Figure 5:**
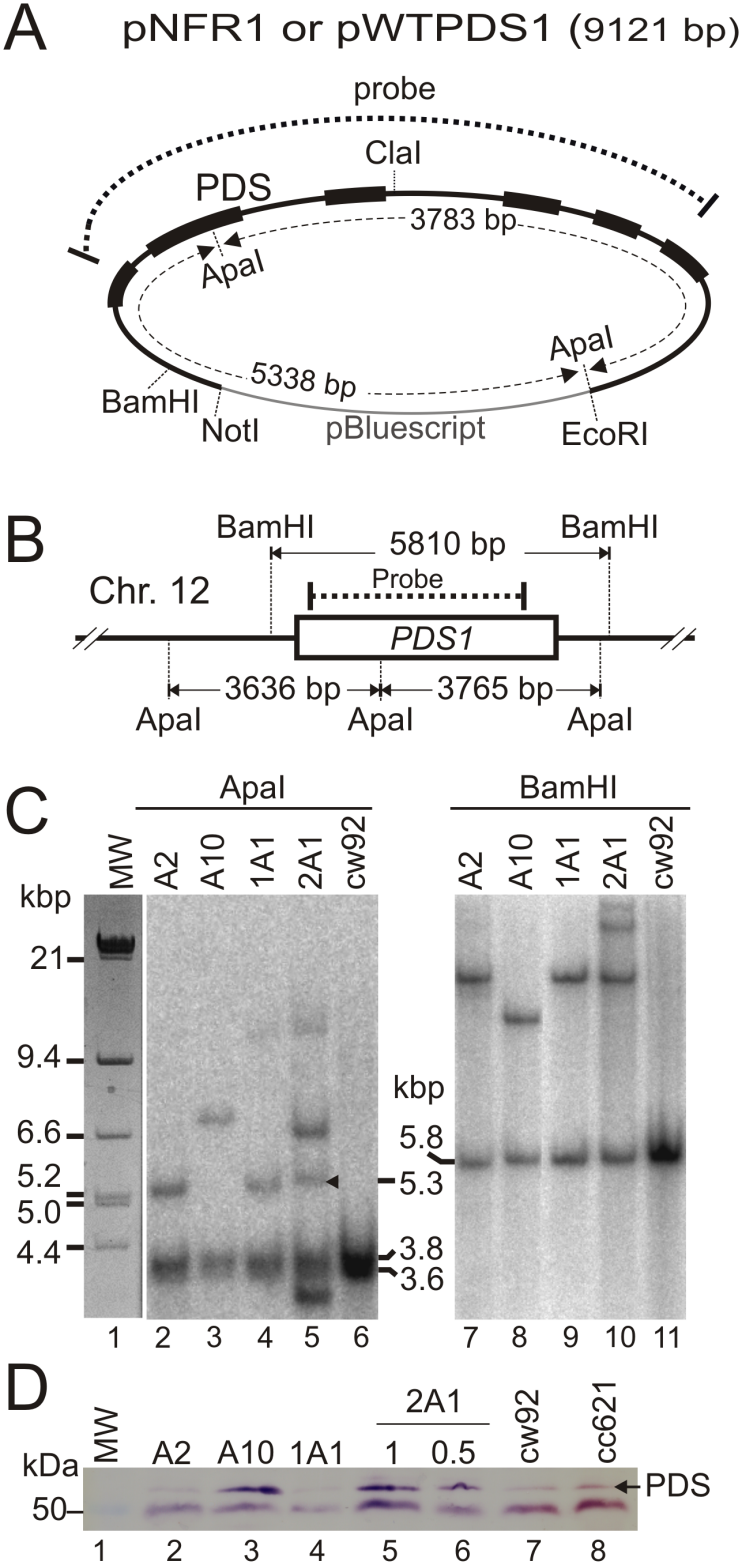
Nuclear transformation vector and analysis of pNFR1 transformants. (**A**) pNFR1 and pWTPDS1 transformation vectors. The two vectors are distinguished by a single base substitution conferring norflurazon resistance in pNFR1. Shown are coding regions (black boxes), pBluescript vector backbone (thin grey line), extent of cDNA probe used for DNA hybridization and restriction enzyme sites. (**B**) Map of the *PDS1* gene (white box) located on chromosome 12 (Chr12). Restriction sites and overlap region of the cDNA probe are shown. (**C**) Blots of digested DNA from pNFR1 transformants and untransformed cw92 strain hybridized with a *PDS1* cDNA probe. Restriction enzyme used, band sizes and MW standards are indicated. (**D**) Protein blot of fractionated total cell protein from pNFR1 cw92 transformants, the cc621 WT strain and the non-transformed cw92 strain incubated with an affinity-purified polyclonal antibody raised against a PDS peptide. The PDS band is arrowed.

To show that the F131V mutation conferred norflurazon resistance, the mutant *pds1-nfr1d* gene was cloned in an *E. coli* plasmid vector and used to transform *C. reinhardtii* cw92, which is a norflurazon sensitive cell-wall deficient strain [28]. The 5′ region of the *pds1-nfr1d* gene was isolated as a 3.2 kbp PCR product (Fig. 4A, primers 1017 and 1114) and joined to the 3′ region of the WT gene amplified as a 3.0 kbp PCR product (Fig. 4A, primers 1113 and 1065). The two cloned PCR products were combined at a *ClaI* site inserted in the third intron and ligated to pBluescript to assemble the 9121 bp pNFR1 plasmid (Fig. 5A). The *ClaI* site was created by inserting an ATC before the GAT trinucleotide at base 130 of the third 612 bp intron. In order to include 5′ and 3′ expression elements, the coding region was flanked by 1468 bp of upstream and 1390 bp of downstream sequences. As a control construct, the equivalent 5′ region was cloned from the *PDS1^WT^* gene and fused to the 3′ end of the *PDS1^WT^* gene to produce the pWTPDS1 plasmid. The only difference between the pNFR1 and pWTPDS1 vectors was the thymine to guanine transversion in exon 2 of the *pds1-nfr1d* gene (Fig. 4A) in the pNFR1 vector, which was confirmed by sequencing both plasmids.

pNFR1 and pWTPDS1 were used to transform the cell wall deficient and norflurazon sensitive strain cw92 using the glass-bead vortexing method [29]. Herbicide resistant colonies were isolated by direct selection on 5 µM norflurazon medium, followed by several rounds of continued selection on 5 µM norflurazon media. We found selection on 2.5 µM norflurazon gave rise to a high background of surviving non-transformed cells. For pNFR1, 45 to 60 herbicide resistant colonies were isolated using 2 µg of supercoiled plasmid with ∼1.5×10^7^ cells. Sixty percent of the colonies isolated on primary selection plates were PCR positive for the pNFR1 plasmid (not shown). In pWTPDS1 or control no-plasmid transformation experiments, typically 0–15 herbicide resistant colonies were obtained with ∼1.5×10^7^ cells on primary selection plates. PCR positive herbicide resistant colonies were only obtained following transformation with pNFR1 and none were isolated using pWTPDS1. The majority of PCR negative colonies from pNFR1 transformation experiments, and colonies from the pWTPDS1 and no plasmid DNA transformation experiments isolated on primary selection plates, grew relatively slowly and died following continued rounds of selection. Four norflurazon resistant colonies transformed with pNFR1 were chosen for further analysis.

### Molecular Analyses of Transgenic pNFR1 Strains

DNA blots hybridized with a *PDS1* cDNA probe are shown in Fig. 5C. The pNFR1 vector contains two *Apa* I sites and one *Bam* HI site (Fig. 5A). The *Apa* I site downstream of the *Eco* R1 site is located in the vector polylinker. *Apa* I digests of non-transformed cw92 DNA gave rise to a broad band comprised of closely migrating 3.6 and 3.8 kbp fragments (Fig. 5C lane 6, see map Fig. 5B). Transformant lanes 2–5 (Fig. 5C) contained additional *Apa* I bands consistent with integration of pNFR1 into the genome. A 5.3 kbp *ApaI* band common to three transformant lanes (Fig. 5C) represents a pNFR1 derived fragment (Fig. 5A). Additional *Apa I* bands in transformant lanes not corresponding to the pNFR1 bands of 3.8 kbp and 5.3 kbp are likely to represent junction fragments. *Bam HI* digests of DNA from all four transformants (Fig. 5C, lanes 7–10) contain the 5.8 kbp WT band (Fig. 5C, lane 11). The transformant lanes contained additional higher MW bands corresponding to integration of pNFR1 into their genomes. Three transformant lanes (A2, A10, 1A1) contained one additional higher MW Bam HI band of similar intensity to the 5.8 kbp BamH1 band corresponding to the single copy endogenous *PDS1* gene. This is consistent with single copy insertions. Transformant 2A1 appeared to contain multiple insertions giving rise to three additional bands in *Bam* HI digests (Fig. 5C, lane 10). Transforming DNA integrates by illegitimate recombination into the nucleus of *C. reinhardtii* and would account for differences in the banding pattern between lanes.

Accumulation of the PDS protein in transformants was compared to non-transgenic strains cw92 and cc621 by protein blot analysis (Fig. 5D). A PDS-specific antibody showed variable accumulation of a PDS band in the four transgenic lines. Transgenic strains A10 and 2A1 accumulated over two-fold higher levels of PDS protein (Fig. 5D, lanes 3, 5 and 6) than the untransformed cw92 and cc621 strains (Fig. 5D, lanes 7–8). Transgenic strains A2 and 1A1 (Fig. 5D, lanes 2 and 4) accumulated similar levels of PDS compared to the non-transgenic cw92 and cc621 strains. A∼50 kDa band present in all lanes serves as a loading control and indicates similar amounts of protein were loaded per lane. Differences in PDS levels between transformants can be explained by position effects resulting from random integration of foreign genes into the *C. reinhardtii* nuclear genome by illegitimate recombination.

### Transgenic pNFR1 Strains were Highly Resistant to Norflurazon

**Figure 6:**
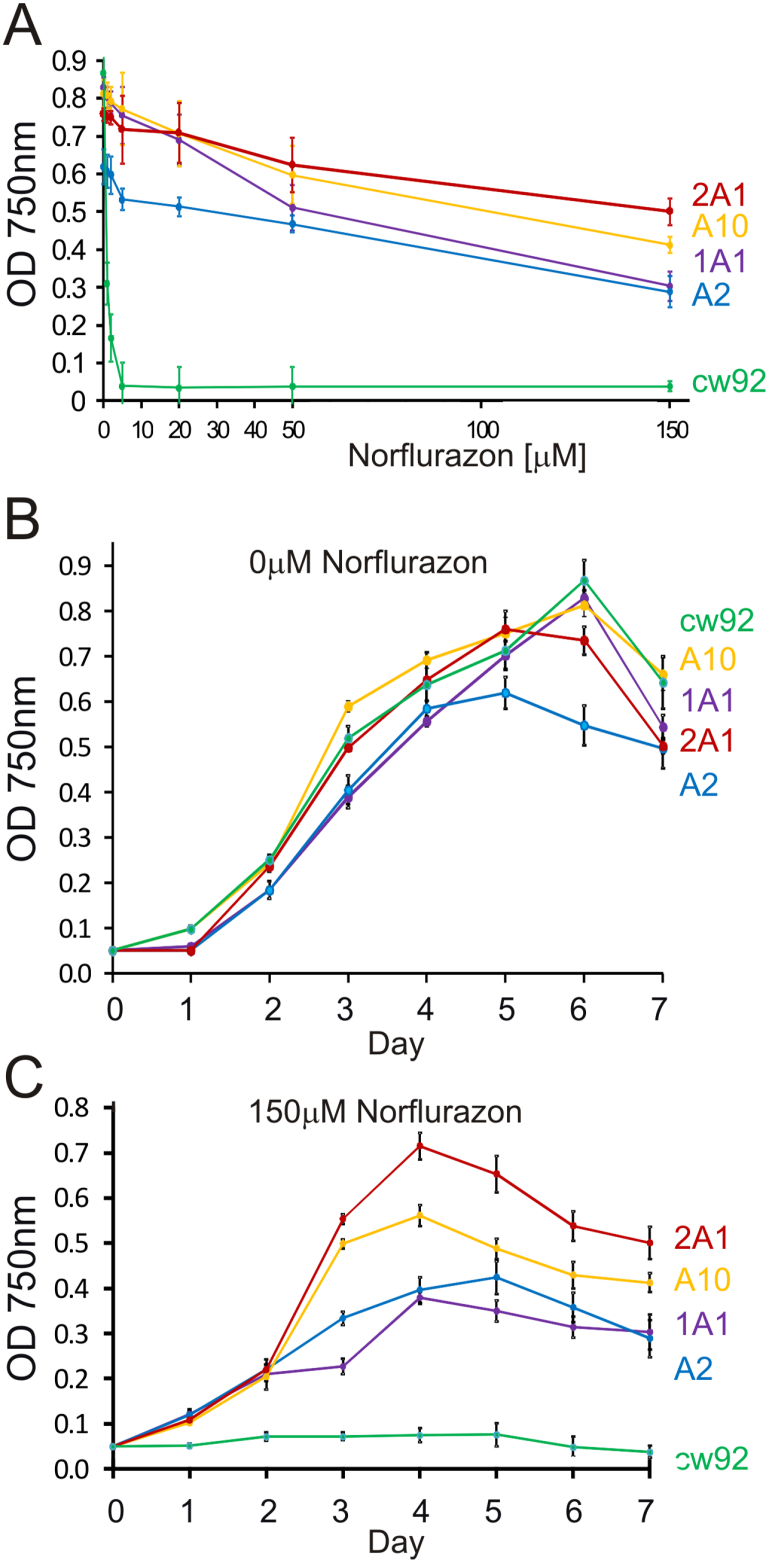
Resistance levels of pNFR1 transformants to norflurazon. Cells were inoculated at an initial OD750 of 0.05 and were grown in TAP media with varying concentrations of norflurazon. (**A**) Norflurazon resistance of cw92 (norflurazon sensitive non-transformed strain) and pNFR1 A2, A10, 1A1 and 2A1 transformants. Optical density was measured after 7 days of growth. (**B–C**) Growth curves of NFR1 cw92 transformants and non-transformed cw92 strain in liquid TAP media without norflurazon (**B**) or with 150 µM norflurazon (**C**). Green line: cw92; blue line: A2; yellow line: A10; purple line 1A1; red line: 2A1. The mean values of three cultures were plotted. Standard error bars are shown.

Growth of pNFR1 transformants and the recipient non-transgenic cw92 strain were compared in TAP media containing increasing concentrations of norflurazon (Fig. 6A). Growth of cw92 was severely reduced by 1 µM norflurazon (67% reduction) and prevented by 5 µM norflurazon after seven days growth. The growth of all transformants showed marked resistance to norflurazon. The highest levels of resistance were shown by strains 2A1 and A10 (Fig. 6A), which accumulated more PDS protein (Fig. 5D). All transgenic strains grew in media containing 150 µM norflurazon. When grown in TAP media without herbicide, the transformants grew at similar rates to WT (Fig. 6B). This indicates that the expression of *pds1-nfr1d* in transgenic strains does not have a marked impact on cell growth. Growth of transformant A2 plateaued earlier than the other strains and reached a final density that was 70% of the WT density at day six. The reduced cell density observed for one transformant (transformant A2) relative to cw92 is likely to reflect factors that are not directly related to the presence of transgenic DNA containing *pds1-nfr1d*, such as the transgene integration site. In the presence of 150 µM norflurazon, transformant 2A1 grew to the highest densities (Fig. 6C).

### F131 Maps to a Rossmann-like Domain and Clusters with other Norflurazon-resistant Mutations in 3D Protein Models

**Figure 7:**
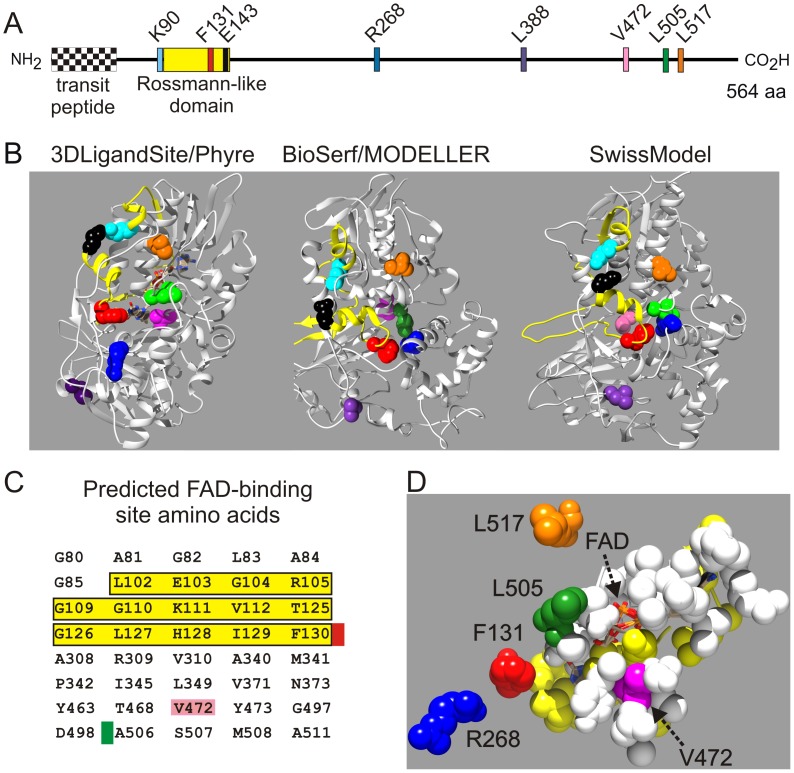
Location of norflurazon resistance mutations on the PDS protein. (**A**) Primary structure of *C. reinhardtii* prePDS protein showing plastid transit peptide, dinucleotide binding Rossmann-like domain, and amino acid substitutions affecting PDS function (see Fig. 8). The same colour coding is used throughout Figs 7 and 8. (**B**) Ribbon representation of the predicted three-dimensional structure of *C. reinhardtii* PDS. Left: Phyre-based 3DLigandSite model; middle: Modeller-based BioSerf predicted structure; right: SwissModel predicted structure. Amino acids shown in A are indicated. The conserved Rossmann-like domain is shown as a yellow ribbon. (**C**) Residues in the predicted FAD-binding domain. Amino acids in the Rossmann-like domain are shaded yellow. Amino acids conferring norflurazon resistance lie adjacent to (F131/red, L505/green) or are part of the predicted domain (V472/pink). (**D**) 3D structure of the predicted FAD-binding region. Bound FAD is shown in the centre of the structure as a ball stick model. Yellow atoms are part of the Rossmann-like domain. F131, R268, V472, L505 and L517 are shown.

**Figure 8:**
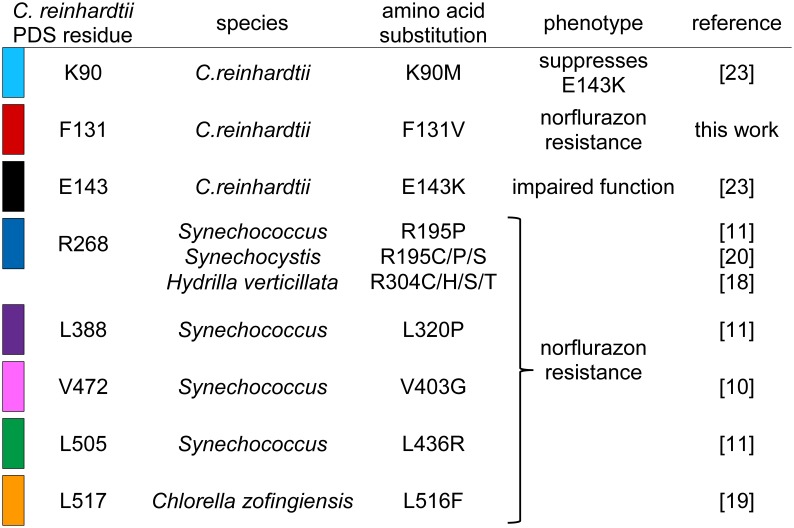
Location of mutations on the *C. reinhardtii* PDS sequence. Shown are amino acid residue numbers in *C.reinhardtii* PDS, species in which the missense mutation was isolated, numbering of the original substitutions in homologous PDS proteins and phenotype of the substitutions. Synechococcus PCC7942 and Synechocystis PCC 6803 are listed. Colour coding of amino acids is the same as in Figure 7.

A linear scheme of the 564 amino acid precursor PDS protein from *C. reinhardtii* containing the chloroplast transit peptide is shown in Figure 7A. F131V and previously described resistance mutations in homologous PDS proteins identify amino acids that are likely to affect norflurazon binding. The high conservation amongst PDS proteins allowed us to map substitutions conferring norflurazon resistance in homologous proteins (Fig. 8) onto the *C. reinhardtii* PDS sequence (Fig. 7A). The F131V mutation reported here lies in the N-terminal quarter, whereas previously reported amino acids substitutions conferring norflurazon-resistant mutations map closer to the middle (R268, L388) or C-terminus (V472, L505, L517) of the 564 amino acid protein (Fig. 7A). The F131V is the first herbicide resistance mutation localising to the dinucleotide binding Rossmann-like domain [30], [31], [32] spanning amino acids Y91 to L144 (Fig. 7A). This organisation of Rossmann-like domain amino acids contributing to a FAD-binding domain is characteristic of pyridine nucleotide-disulphide oxidoreductases [31]. A previously described substitution (E143K) in this domain reduces function and is suppressed by intragenic suppressor mutation K90M [23].

The crystal structure of a plant PDS has not been determined. Whilst the structure of a CRTI-type PDS enzyme present in bacteria and fungi is available [33], it is distantly related to the plant PDS enzyme and may have an independent evolutionary origin [34]. In order to map the F131V mutation and the positions of other norflurazon resistance mutations onto the structure of PDS, 3D models of the *C. reinhardtii* protein were constructed using four programs: 3DLigandSite [35] which uses the Phyre prediction system [36], MODELLER [37], [38] via the BioSerf service [39], SwissModel [40] (Fig. 7B), and 3D-Jigsaw [41]. The predicted PDS structures are shown in Figure 7B and Figure S1. Each program used the crystal structures of different proteins to predict the folded structure of PDS. Phyre used human monoamine oxidase type B [42], MODELLER used an oxidoreductase from *Methanosarcina mazei* [43] and SwissModel used putrescine oxidase from *Arthrobacter aurescens* [44]. *C.reinhardtii* PDS shares: 15% identity (32% similarity) with human monoamine oxidase chain B over aligned PDS amino acids 76–537; 16% identity (30% similarity) with *M. mazei* oxidoreductase for PDS amino acids 76–526; 18% identity (32% similarity) with *A. aurescens* putrescine oxidase for PDS amino acids 76–553.

In all four models of the folded protein, three amino acids implicated in norflurazon resistance F131 (red), V472 (pink) and L505 (green), cluster together in the protein (Fig. 7B, Fig. S1). R268 (blue), whose substitution confers norflurazon resistance in cyanobacteria and *Hydrilla* (Fig. 8), is also part of the cluster in the 3D structures predicted by MODELLER, SwissModel (Fig. 7B) and 3-DJigsaw (Fig. S1). In the 3D structure predicted by Phyre, R268 lies outside the cluster in close proximity to F131 (Figs. 7B and 7D). Mutations of amino acids L388 (purple) and L517 (orange) also confer norflurazon resistance (Fig. 8). L388 and L517 are located on opposite sides of the cluster (Fig. 7B). Previous work using 3D–LigandSite has suggested E143 (black) and K90 (cyan) are juxtaposed in the folded protein [23]. Consistent results were obtained with Bioserf/MODELLER, SwissModel (Fig. 7B) and 3D–Jigsaw [41], [45] (Fig. S1), where E143 and K90 lie close to each other in the predicted protein structures. Amino acids in the Rossmann-like domain (Y91-L144) are shown as a yellow ribbon in Figure 7B. Whilst both F131 (red) and E143 (black) are located in the Rossmann-like domain (Fig. 7A), they are separated in the folded protein models (Fig. 7B).

Norflurazon inhibits PDS activity by competing with the enzyme’s cofactors rather than the phytoene substrate [46]. The purified plant enzyme is a flavoprotein containing FAD [47]. In bacterial CRT-I type PDS, FAD appears to be the only co-factor required for phytoene desaturation [33]. Following the desaturation reaction the reduced FADH_2_ produced is re-oxidised to FAD by plastoquinone either directly or indirectly [48]. The 3DLigandSite program [35] predicts the location of the FAD binding domain [32], [49], [50] in PDS. The amino acids contributing to the predicted FAD binding domain in the *C. reinhardtii* PDS enzyme are shown in Fig. 7C. These include amino acids that are also part of the Rossmann-like domain (Fig. 7C, shaded yellow). Of the amino acids whose substitution confers norflurazon resistance, V472 (pink) is part of the predicted FAD-binding domain, whilst F131 (red) and L505 (green) lie adjacent to FAD-binding domain residues F130 and A506 (Fig. 7C). A magnified view of the relative positions of F131, V472 and L505 and FAD in the 3D-ligand model of PDS is shown in Figure 7D. The close proximity of F131, V472 and L505 to the predicted FAD binding site is consistent with norflurazon inhibiting the function of this co-factor.

### Cross-tolerance Conferred by the *pds1-nfr1d* Gene to other Bleaching Herbicides

**Figure 9:**
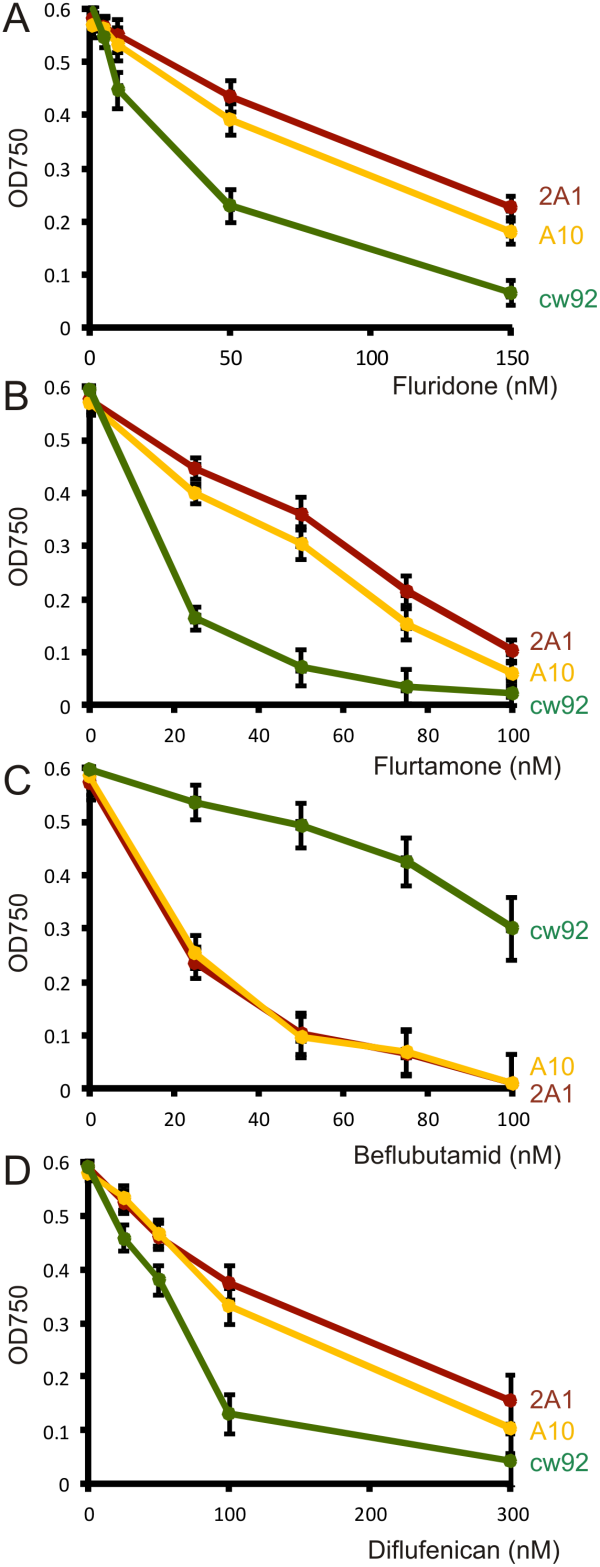
The effect of bleaching herbicides on growth of cw92 (non-transformed strain) and pNFR1 transgenic lines. (**A**) Fluridone, (**B**) Flurtamone, (**C**) Beflubutamid, and (**D**) Diflufenican. Cells were inoculated at an OD750 of 0.05 at day 0 in TAP media and cell density estimated by OD750 on day 7. Values represent the average of three replicate cultures. Standard error bars are shown. Red line: strain 2A1; yellow line: strain A10; green line: cw92.

The highest levels of norflurazon resistance were exhibited by pNFR1 transformants 2A1 and A10. Four different bleaching herbicides were tested on these transformants: fluridone, flurtamone, beflubutamid and diflufenican (Figs. 9A–D). Wild-type cells showed a marked reduction in growth when placed in TAP medium containing 50 nM fluridone (Ec50 = 38 nM), 25 nM flurtamone (Ec50 = 16 nM) and 100 nM diflufenican (Ec50 = 68 nM). The WT strain was relatively tolerant to beflubutamid and grew at all concentrations tested (Ec50>100 nM). Transformants 2A1 and A10 showed greater tolerance to fluridone, flurtamone and diflufenican at all the concentrations tested. The average Ec50 values for the two transformants were: 110 nM for fluridone, 56 nM for flurtamone, and 170 nM for diflufenican. In contrast, pNFR1 transformants exhibited enhanced sensitivity to beflubutamid (Ec50 = 21 nM) with resistance levels 4.8 fold lower than wild type cells (Ec50>100 nm).

## Discussion

We used forward genetics to isolate a new F131V substitution in the *C. reinhardtii* PDS protein that confers a high level of resistance to norflurazon. Whilst growth of WT cells was prevented by 5 µM norflurazon, cells containing the mutant *pds1-nfr1d* gene were able to grow in media containing 150 µM norflurazon. The Ec50 values to norflurazon were 0.7 µM and around 100 µM, for WT and mutant NFR1 cells, respectively. The norflurazon-resistance mutation conferred cross-tolerance to the bleaching herbicides fluridone, flurtamone, and diflufenican but enhanced sensitivity to beflubutamid, relative to WT cells. The mutant *pds1-nfr1d* allele is dominant or semi-dominant to the WT gene allowing the cloned *pds1-nfr1d* gene to be used as a marker for nuclear transformation with direct norflurazon selection for resistant transgenic cells.

The F131V substitution is the first norflurazon resistance mutation to be mapped to the dinucleotide binding Rossmann-like domain of PDS. F131V and three other resistance mutations, which correspond to substitutions at R268, V472 or L505 in the *C. reinhardtii* protein (Fig. 8), are located at distant sites of the primary sequence but cluster together in the predicted tertiary structure. Moreover, three of these amino acids lie adjacent to (F131, L505) or are part of (V472) the predicted FAD binding domain (Fig. 7C). These results, based on locating amino acid substitutions conferring norflurazon resistance on 3D-models of PDS, predict the herbicide’s target region on the protein and suggest the herbicide’s mode of action involves inhibition of FAD function. Substitutions of amino acids L388 and L517 also confer norflurazon resistance but these residues do not map close to the F131-R268-V472-L505 cluster of resistance mutations on 3D models. To resolve whether L388 and L517 interact with norflurazon directly or affect binding of norflurazon indirectly by affecting the folding of the protein will become clearer if a crystal structure becomes available.

Use of the *pds1-nfr1d* allele as a positive selection marker for nuclear transformation of *C. reinhardtii* avoids the use of antibiotic resistance genes [51], [52], [53], [54], [55], which may raise regulatory concerns for commercial applications of transgenic algae. The transformation procedure used the mutant *pds1-nfr1d* allele with its own promoter and terminator and 5 µM norflurazon, which was substantially higher than the concentration required to kill the majority of non-transformed cells (Ec50 of 0.7 µM). In a recent study, 0.5 µM norflurazon was used to select *C. reinhardtii* transformants expressing a cDNA encoding a mutant PDS protein in which the L505F substitution was introduced by site-directed mutagenesis [24]. The RbcS2 promoter [52] was used to express the L505F mutant gene resulting in elevated PDS transcripts [24]. Here we found that 5 µM norflurazon was required for effective selection of transformants in the light regimes used. Use of 2.5 µM norflurazon on selection plates led to a high background of non-transformed colonies. Previous herbicide resistant marker genes [24], [56] and a mutant CRY1 gene providing resistance to the translational inhibitors emetine and cryptopleurine [57] have used 5′ and 3′ regulatory elements from the RbcS2 gene to allow the isolation of nuclear *C. reinhardtii* transformants [24], [56], [57]. The use of herbicide resistance genes with native regulatory elements [58] including the mutant *pds1-nfr1d* gene (this work) as marker genes overcomes the possibility of *RbcS* gene silencing and titration of transcription or translation factors needed to express the native *RbcS* genes [59] in *C. reinhardtii*.

Transformants containing the *pds1-nfr1d* gene were 4.8 fold less resistant to beflubutamid than WT cells. By comparison, transformation of the WT *Hydrilla* PDS protein into Arabidopsis increased seedling sensitivity to diflufenican [18]. Arabidopsis seedlings expressing a *Hydrilla* norflurazon resistant PDS protein with a R304C (corresponds to position R268 in the *C. reinhardtii* protein) mutation, were more sensitive to both diflufenican and beflubutamid [18]. These results show that changes in the region identified by the cluster of amino acid substitutions conferring norflurazon resistance (Figs. 7B, 7D) are critical in determining the action of herbicides and provide a step towards the rational design of new herbicides targeting this protein. The enhanced susceptibility of *pds1-nfr1d* transformants to beflubutamid allows negative selection. This enables *pds1-nfr1d*, which is a native algal gene, to be used for either positive or negative selection, which is conditional on the selection agent used. Norflurazon enables positive selection whereas beflubutamid allows negative selection. Because the transformants contain both the WT and norflurazon-resistant PDS enzymes, this suggests that beflubutamid sensitivity is dominant or semi-dominant to resistance conferred by the WT enzyme. Raised PDS levels in the A10 and 2A1 transformants relative to WT cells was consistent with higher levels of the beflubutamid-sensitive mutant PDS enzyme in transgenic cells.

Dual and conditional positive/negative selection markers are powerful tools for manipulating genomes and controlling the growth of transgenic cells [60], [61], [62]. The high norflurazon resistance levels exhibited by *pds1-nfr1d* transformants suggests the coding region flanked by appropriate regulatory elements may serve as a marker for transformation of a wide range of algae and plants susceptible to norflurazon and related bleaching herbicides. The PDS protein is localised to chloroplasts, which raises the possibility of using a modified coding sequence with chloroplast regulatory elements as a new marker gene for the chloroplast transformation toolbox [63]. Negative selection can be used to restrict the spread of transgenic cells, influence recombination events such as marker excision, and in mutant screens. The *pds1-nfr1d* allele confers sensitivity to beflutamid and is a candidate for a new negatively selectable marker for algal genetics.

## Conclusions

A phenylalanine to valine substitution at position 131 in the dinucleotide binding Rossmann-like domain of phytoene desaturase increased the 5 µM norflurazon concentration needed to restrict growth of WT *C. reinhardtii* cells to over 150 µM in mutant cells. The F131V substitution clusters with two other substitutions conferring norflurazon resistance close to the predicted FAD-binding site in 3D-protein models.

## Materials and Methods

### *C. reinhardtii* Strains, Media, Mutagenesis and Genetic Crosses

All *C. reinhardtii* strains were from the Chlamydomonas Center (University of Minnesota). Strains were maintained at 20°C to 25°C on TAP medium [1]. Transformants were propagated on TAP medium with 5 µM norflurazon (Sigma-Aldrich, Poole, UK). Large 25 ml–200 ml liquid cultures were grown at 25°C in a New Brunswick Scientific Innova 4340 illuminated orbital shaker at 120 rpm. Arginine (Sigma-Aldrich) was used at a final concentration of 50 mg L^−1^ and nicotinic acid (Sigma-Aldrich) at 25 mg L^−1^.

For mutagenesis 5 ml of 1×10^7^ cells ml^−1^ of *C. reinhardtii* strain *nic7 arg7 y1* mt*+* were irradiated for 20 seconds with a 254 nm XX-15 BlackRay germicidal UV lamp (UVP, Upland, California) at 2.42 mW cm^−2^ in 9 cm petri dishes with gentle rocking. Irradiated cells were kept in the dark for 12 h, and then concentrated by centrifugation at 600×g at room temperature. Three norflurazon resistant cell lines (NFR1-3) were selected on media containing 33 µM norflurazon at 25°C with a light intensity of 60 µmol m^−2 ^s^−1^ in a 12 h light cycle. Only NFR1 survived several rounds of serial propagation on 33 µM norflurazon selective plates. Two additional resistant mutants were isolated using UV irradiation of 2.86 and 3.62 mW cm^−2^ for 20 seconds but these grew very slowly and could not be propagated.

Genetic crosses to isolate vegetative diploid cells or zygotes giving rise to meiotic tetrads for segregation analyses were performed as described [1], [64]. Norflurazon resistant strain NFR1a (*arg7, NIC7, pds1-nfr1d*, mt+) was isolated from a meiotic product of the cross NFR1 (*nic7, arg7, pds1-nfr1d,* mt+) with an *arg2* mt− strain. NFR1a was sensitive to paraquat. Diploids were made by crossing paraquat-resistant strain PQR20 (ARG7, *nic7*, *PDS1*, *pqr* mt−) with NFR1a; the *pqr* locus responsible for paraquat resistance has not been identified. PQR20 was sensitive to norflurazon. Eighty one diploid colonies capable of growing on media without arginine or nicotinic acid were resistant to both norflurazon (33 µM) and paraquat (39 µM). NFR1 (*nic7, arg7, pds1-nfr1d*, *y1* mt+) was crossed with either S1D2 mt− [65] or CC621 mt− to provide tetrads for segregation analysis.

### Nucleic Acid Methods

Total DNA from *C. reinhardtii* was prepared according to Day et al 1988 [66], except an additional DNA precipitation was included by adding 0.6 volumes of 20% PEG 8000, 1.5 M NaCl (Sigma-Aldrich) to the total DNA solution. RNA was purified using the RNeasy plant mini kit (Qiagen) according to the manufacturer’s instructions. Genomic DNA for PCR screening was isolated according to Cao *et al.* (2009) [67]. Hybridisation probes were comprised of a Gulliver [26] internal fragment and a 1.5 kbp *PDS1* cDNA from *C. reinhardtii* strain CC-503 cw92 mt+ prepared using nested RT-PCR with primers 1297 and 1298 followed by primers 1328 and 1329. Probes were labelled with [α-^32^P] dCTP (PerkinElmerNEN, Seer Green, UK) using the High Prime labelling kit (Roche, Mannheim, Germany). Digests of 2 µg of total DNA digests were fractionated on 0.7% W/V agarose gels for DNA blot analysis [66]. Membranes were washed at 65°C in 0.1×SSC buffer and 0.1% w/v SDS. Table S1 lists the oligonucleotides used. PCR screening of transformants was performed using primer pairs 1322+1323, and 1324+1325, which amplified the regions spanning the PDS-vector junctions in pNFR1 and pWTPDS1 transformants. PCR reactions with actin gene primers 1127 and 1351 were used as positive controls to verify the quality of genomic DNA. PCR screening was performed using BioMix PCR master mix (Bioline, London). BigDye terminator v3.1 (Applied Biosystems) was used for cycle sequencing and the ladders fractionated using a 48 capillary ABI 3730 DNA Analyser.

### Isolation and Sequence Analysis of the *pds1-nfr1d* Gene

PCR products from NFR1 genomic DNA using Vent DNA polymerase (New England Biolabs, Massachusetts, USA) were sequenced directly or following cloning into pGEM-T Easy (Promega, Madison, USA). Direct sequencing was on: (i) a 1.3 kbp product containing exons 1 and 2 (PCR primers 889 and 891; sequencing primers 870, 871, 890 and 892); (ii) a 470 bp product containing exon 3 (PCR primers 872 and 873; sequencing primers 873 and 874); (iii) a 462 bp product containing exon 4 (PCR primers 875 and 876; sequencing primers 876 and 877); (iv) a 662 bp product containing exons 5 and 6 (PCR primers 878 and 879; sequencing primers 879 and 880). A 3235 bp PCR product containing exons 1–3 was amplified with primers 1017+1114, cloned into pGEM-T and sequenced.

### Construction of pNFR1 and pWTPDS1

The pWTPDS1 plasmid was constructed by joining 5′ (3235 bp; primers 1017+1114) and 3′ (2965 bp; 1113+1065) PCR products (cloned in pGEM-T Easy) amplified from CC503 cw92 mt+ total DNA via a *Cla* I site located in primers 1113 and 1114. The WTPDS1 sequences in pGEM-T Easy were excised with *Not I* and *Cla I* (3235 bp sequence) and *Cla I* and *EcoR1* (2965 bp sequence) and cloned into pBluescript KS- [68]. To construct pNFR1, the 3.2 kbp 5′ WT PDS1 *Not* I - Cla I fragment was replaced with the equivalent 5′ *pds1-nfr1d* fragment. The complete inserts in both plasmids were sequenced to exclude PCR errors.

### Nuclear Transformation of *C. reinhardtii*

Chlamydomonas cell wall deficient strain CC-503 cw92 mt+ was transformed according to Kindle, 1990 [29]. 25 ml of cell culture (∼5×10^6^ cells/ml) was sedimented at 500×g. The cell pellet was resuspended in 2.5 ml of TAP media and 300 µl mixed with 0.3 g of sterile 0.4 mm glass beads (BDH, Poole, UK), 2 µg circular (mainly supercoiled) plasmid DNA and 100 µl of 20% (W/V) polyethylene glycol (M_W_ 8000). The mixture was vortex-mixed (Rotamixer, *Hook and Tucker Instruments* Ltd., Croydon, UK) at maximum setting for 30 seconds, 700 µl of TAP media was added, and the cells spread onto three 9 cm selection plates containing solid (1% W/V agar) TAP media with 5 µM norflurazon. Cells were allowed to recover for 36 h in dim light (10 µmol m^− 2 ^sec^−1^) and then transferred to high-light (250 µmol m^−2 ^sec^−1^). Colonies became visible after 21 days and were transferred to fresh selection media.

### The Effects of Herbicides on Cell Cultures

Growth curves for the NFR1 strain were prepared by inoculating cells to a concentration of 1×10^5^ cells ml^−1^, growing them for 7 days in 50 ml of TAP media in a 12 h light cycle (60 µmol m^−2 ^s^−1^) at 25°C with shaking (120 rpm) with increasing concentrations of norflurazon and measuring optical density (OD) at 750 nm. Transformant herbicide growth assays were carried out in continuous light (260 µmol m^−2 ^s^−1^) at 25°C without shaking and culture ODs measured daily in a microplate reader (Biotek Synergy, Potton, UK). Transformant cells were inoculated in 1 ml of TAP media to an OD750 nm of 0.05 containing increasing concentrations of norflurazon in 24-well plates and grown for 7 days. Resistance curves for other herbicides were obtained by growing cells in 200 µl TAP media with increasing concentrations of herbicides in 96-well plates. Fluridone was used at 5, 10, 50 and 150 nM concentrations. Befublutamid and flurtamone were utilised at 25, 50, 75 and 100 nM concentrations. Diflufenican was used at 25, 50, 100 and 300 nM concentrations. All experiments were performed in triplicate. Herbicides were from Sigma-Aldrich.

For estimation of coloured carotenoids, cultures were grown for 7 days in TAP media in a 12 h day (60 µmol m^−2 ^s^−1^) with shaking (120 rpm) at a starting density of 1×10^5^ cells ml^−1^. Carotenoids in sedimented cells (1.1×10^8^ cells) were extracted twice with 4 ml of 80% acetone for 20 minutes. Carotenoids were partitioned into 8 ml of 50% diethyl ether with the addition of 2 M NaCl to promote phase separation. The organic phase was washed twice with 5 ml of 4 M NaCl and then dried under nitrogen. The dried carotenoids were dissolved in diethyl ether and coloured carotenoids were estimated from the absorbance at 470 nm [1].

### Protein Blot Analyses

Cells were sedimented from 50 ml cultures at 1×10^7^ cells ml^−1^, frozen, resuspended in 300 µl of 1×SSC (0.15 M NaCl, 0.015 M Trisodium citrate) and 100 µl 4×gel sample loading buffer added (0.25 M Tris-HCl, pH 6.8, 40% V/V glycerol, 12% W/V SDS, 20% V/V β-mercaptoethanol, 0.4% W/V bromophenol blue). The mixture was placed in a boiling water bath for 5 minutes and then sedimented for 2 min (2000 rpm, in an Eppendorf 5415C microfuge). For SDS-PAGE, 6 µl of the supernatant was loaded per lane on 10 well, 12% (W/V) polyacrylamide gels with 4% stacking gels and electrophoresed in Tris-Glycine SDS buffer using the MiniProtean II system (BioRad, Hemel Hempsted, UK). Proteins were electroblotted onto Hybond ECL nitrocellulose (GE Healthcare) and incubated with antibody as previously described [69]. An affinity-purified polyclonal antibody raised in rabbits against peptide VAAWKDEDGDWYETG (Eurogentec, Belgium) was used to detect PDS. The primary antibody was detected with an anti-rabbit IgG-alkaline phosphatase conjugate (Sigma-Aldrich, Poole, UK) and visualised using 5-bromo-4chloro-3-indolyl phosphate/nitro blue tetrazolium (NBT/BCIP) reagent (Sigma-Aldrich).

### Models of Protein Tertiary Structure

The *C. reinhardtii* PDS sequence (GI 158279545) was submitted for structural prediction using default parameters to the 3DLigandSite [35], BioSerf [39], SWISS MODEL [40] and 3D-JIGSAW [41], [45]. Protein structure files were visualised and annotated using UCSF Chimera [70]. Images were rendered using POV-Ray within UCSF Chimera.

## Supporting Information

Figure S1 **Space-filling model of** ***C.reinhardtii*** **phytoene desaturase predicted by 3D Jigsaw** [41], [45] **.** Amino acids F131, R268, L388, V472, L505 and L517 whose substitution are associated with norflurazon resistance are shown (see Figs. 7 and 8). Also shown are E143 and K90. Mutation of E143 impairs function and this is suppressed by a mutation at K90 [23]. The F131, R268, V472 and L505 cluster of amino acids associated with norflurazon resistance is magnified on the right.
(TIF)

Table S1 **Sequences of oligonucleotides used as primers for PCR and sequencing reactions.**
(DOCX)
