# Supplementary figures and images for: A New F131V Mutation in Chlamydomonas Phytoene Desaturase Locates a Cluster of Norflurazon Resistance Mutations near the FAD-Binding Site in 3D Protein Models
Source: PLoS One. 2014 Jun 17;9(6):e99894. doi: 10.1371/journal.pone.0099894 (PMC4061028; doi:10.1371/journal.pone.0099894)

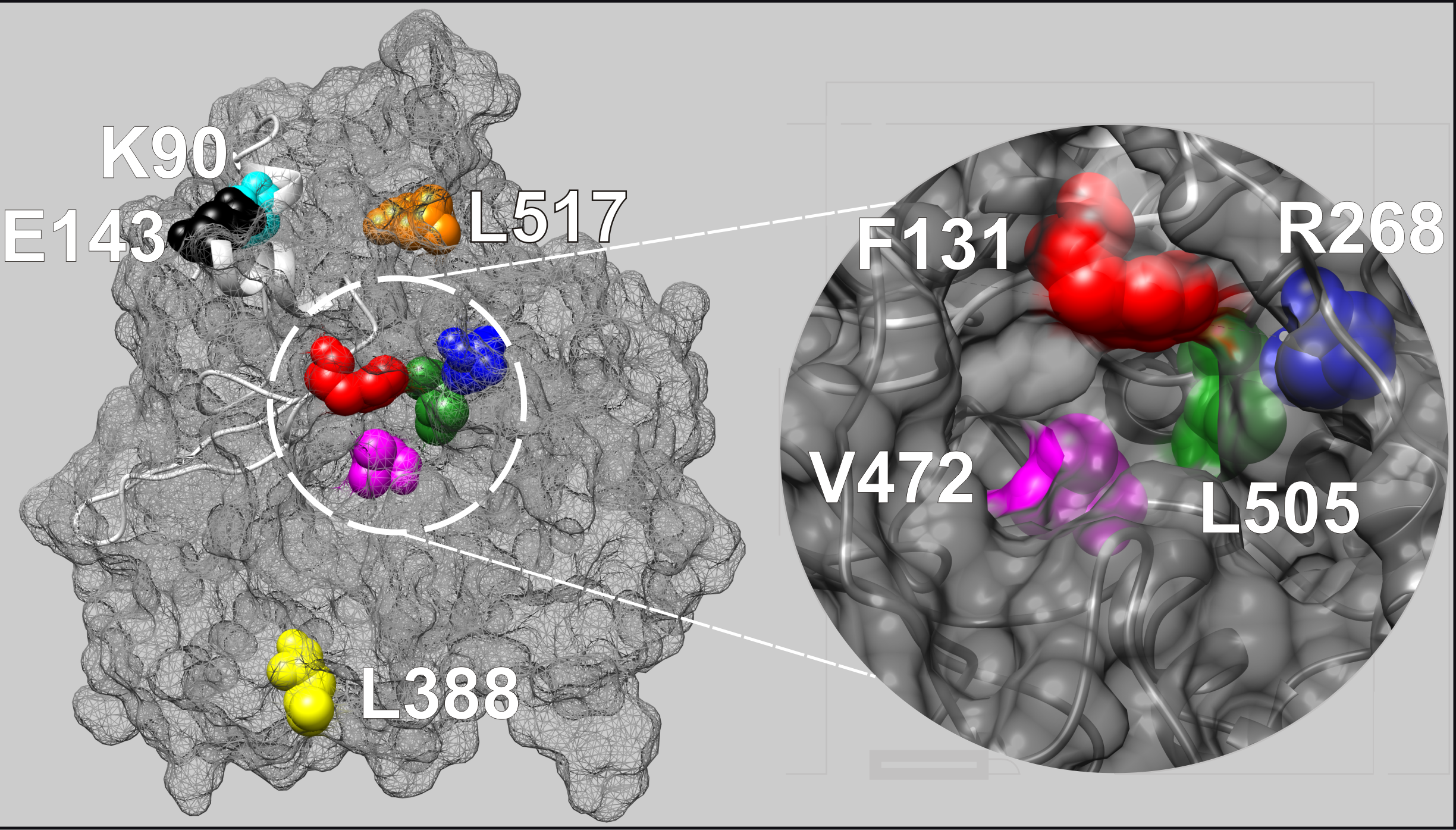

Supplement: Figure S1 — Space-filling model of C.reinhardtii phytoene desaturase predicted by 3D Jigsaw [41], [45] . Amino acids F131, R268, L388, V472, L505 and L517 whose substitution are associated with norflurazon resistance are shown (see Figs. 7 and 8). Also shown are E143 and K90. Mutation of E143 impairs function and this is suppressed by a mutation at K90 [23]. The F131, R268, V472 and L505 cluster of amino acids associated with norflurazon resistance is magnified on the right. (TIF) [file pone.0099894.s001.tif]
