# Supplementary material for: A New F131V Mutation in Chlamydomonas Phytoene Desaturase Locates a Cluster of Norflurazon Resistance Mutations near the FAD-Binding Site in 3D Protein Models
Source: PLoS One. 2014 Jun 17;9(6):e99894. doi: 10.1371/journal.pone.0099894 (PMC4061028; doi:10.1371/journal.pone.0099894)
Supplement: Table S1 — Sequences of oligonucleotides used as primers for PCR and sequencing reactions. (DOCX) [file pone.0099894.s002.docx]

| **Primer no.** | **sequence 5’ → 3’** |
| --- | --- |
| 870 | CACGCCATCTCCTGGTACAATC |
| 871 | CATAAATGGACAATTGGGCTAAC |
| 872 | ACTCAAAGTCATGCCATGGAACTCCC |
| 873 | TCTACCGGCGTCTCAACATGGC |
| 874 | CGCCCCACCCGCCCTGACCC |
| 875 | GGCACGCCCAACAGTCCGACAT |
| 876 | AAGCCTACGTCCGTGCGCCGTG |
| 877 | CCCTAACTCCCGCTCTTCCCTCC |
| 878 | CTGTTCCCCCAGAAAGCTTCTCAAAGCT |
| 879 | TACACGTTTACCGGCCAAAGCC |
| 880 | ACCCCTGCATGTTAGCCTAC |
| 889 | CAGGTAACTGACAATTCAAGTGAGTGGC |
| 890 | CATTTAGAGGGTGGGTCATTTAGCAC |
| 891 | CTGTTTGACCACTGGTGATAACTCTG |
| 892 | CCCAATTGTCCATTTATGCTAC |
| 1017 | AGGTGCGCAGCAAGTGTCAC |
| 1065 | TTTCGCCAGATAGAACCAGCTC |
| 1113 | ATCGATTCCCGCCCGCCGTCCTCCAGT |
| 1114 | ATCGATGGACTCTGGATGGTGGTACGG |
| 1127 | GAGGTCTCTGCTCTGGTGTGCGACAATG |
| 1297 | ATGCAGACCCAGGTCAAGCCCT |
| 1298 | TTCTGCTTGGTGAAGTCGCCCG |
| 1322 | ACGCTTACAATTTCCATTCGCCATT |
| 1323 | CACTTGCTGCGCACCTATCGAATT |
| 1324 | TGCCTCTCAACTGATATCTTTACAGGGC |
| 1325 | CACTTTATGCTTCCGGCTCGTATGTT |
| 1328 | AGGTCAAGCCCTCTAGCTCG |
| 1329 | GTGAAGTCGCCCGCCAGGAA |
| 1351 | CGAGTCCTGCAGAGTCCAAAGAAGCA |
